# Supplementary figures and images for: The Activation of Prothrombin Seems to Play an Earlier Role than the Complement System in the Progression of Colorectal Cancer: A Mass Spectrometry Evaluation
Source: Diagnostics (Basel). 2020 Dec 11;10(12):1077. doi: 10.3390/diagnostics10121077 (PMC7763171; doi:10.3390/diagnostics10121077)

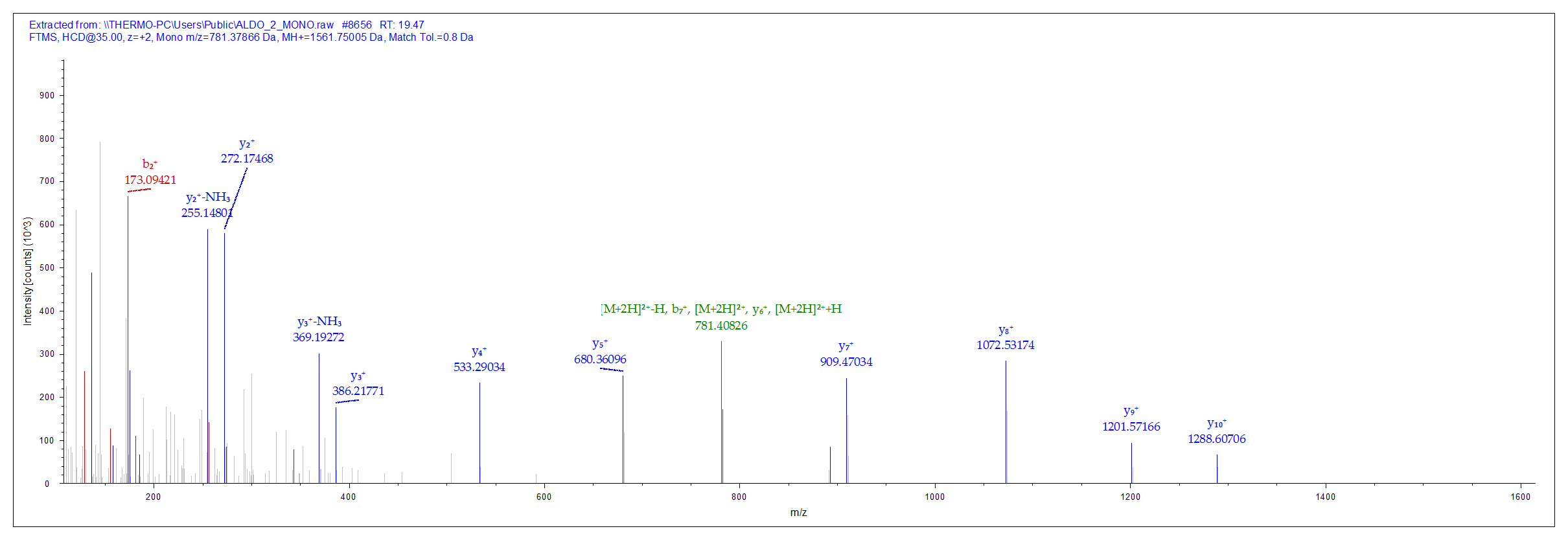

Supplement: Supplementary file 1 [file diagnostics-10-01077-s001.zip › RomanoEtAl_CRC_MS_evaluation_FigureS1.tif]

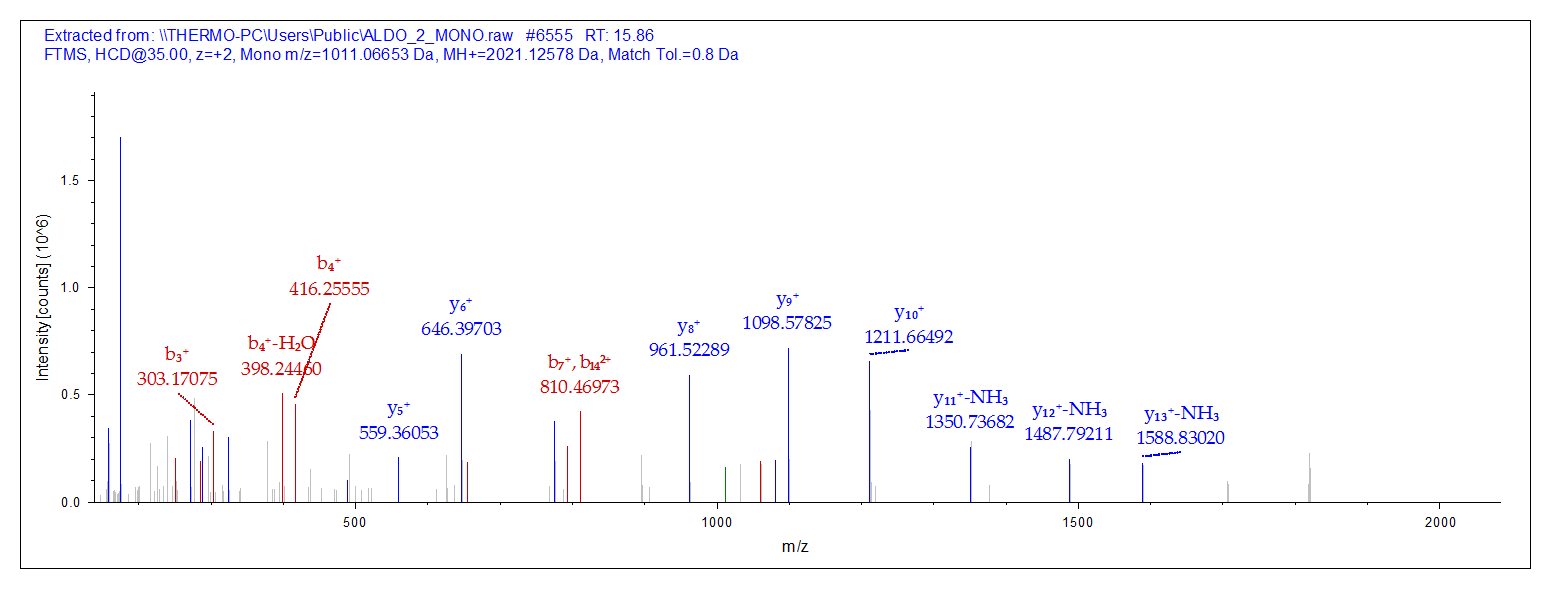

Supplement: Supplementary file 1 [file diagnostics-10-01077-s001.zip › RomanoEtAl_CRC_MS_evaluation_FigureS2.tif]
